# Substitution of Nonpharmacologic Therapy With Opioid Prescribing for Pain During the COVID-19 Pandemic

**DOI:** 10.1001/jamanetworkopen.2021.38453

**Published:** 2021-12-10

**Authors:** Byungkyu Lee, Kai-Cheng Yang, Patrick Kaminski, Siyun Peng, Meltem Odabas, Sumedha Gupta, Harold D. Green, Yong-Yeol Ahn, Brea L. Perry

**Affiliations:** 1 Department of Sociology, Indiana University Bloomington, Bloomington; 2 Luddy School of Informatics, Computing, and Engineering, Indiana University Bloomington, Bloomington; 3 Luddy School of Informatics, Computing, and Engineering, Department of Sociology, Indiana University Bloomington, Bloomington; 4 Department of Economics, Indiana University–Purdue University, Indianapolis; 5 Department of Applied Health Science, School of Public Health, Indiana University Bloomington, Bloomington; 6 Center for Complex Networks and Systems Research, Luddy School of Informatics, Computing and Engineering, Indiana University Bloomington, Bloomington; 7 Network Science Institute, Indiana University Bloomington, Bloomington; 8 Connection Science, Massachusetts Institute of Technology, Cambridge; 9 Department of Sociology, Network Science Institute, Indiana University Bloomington, Bloomington

## Abstract

**Importance:**

During the pandemic, access to medical care unrelated to COVID-19 was limited because of concerns about viral spread and corresponding policies. It is critical to assess how these conditions affected modes of pain treatment, given the addiction risks of prescription opioids.

**Objective:**

To assess the trends in opioid prescription and nonpharmacologic therapy (ie, physical therapy and complementary medicine) for pain management during the COVID-19 pandemic in 2020 compared with the patterns in 2019.

**Design, Setting, and Participants:**

This retrospective, cross-sectional study used weekly claims data from 24 million US patients in a nationwide commercial insurance database (Optum’s deidentified Clinformatics Data Mart Database) from January 1, 2019, to September 31, 2020. Among patients with diagnoses of limb, extremity, or joint pain, back pain, and neck pain for each week, patterns of treatment use were identified and evaluated. Data analysis was performed from April 1, 2021, to September 31, 2021.

**Main Outcomes and Measures:**

The main outcomes of interest were weekly rates of opioid prescriptions, the strength and duration of related opioid prescriptions, and the use of nonpharmacologic therapy. Transition rates between different treatment options before the outbreak and during the early months of the pandemic were also assessed.

**Results:**

A total of 21 430 339 patients (mean [SD] age, 48.6 [24.0] years; 10 960 507 [51.1%] female; 909 061 [4.2%] Asian, 1 688 690 [7.9%] Black, 2 276 075 [10.6%] Hispanic, 11 192 789 [52.2%] White, and 5 363 724 [25.0%] unknown) were enrolled during the first 3 quarters in 2019 and 20 759 788 (mean [SD] age, 47.0 [23.8] years; 10 695 690 [51.5%] female; 798 037 [3.8%] Asian; 1 508 023 [7.3%] Black, 1 976 248 [9.5%] Hispanic, 10 059 597 [48.5%] White, and 6 417 883 [30.9%] unknown) in the first 3 quarters of 2020. During the COVID-19 pandemic, the proportion of patients receiving a pain diagnosis was smaller than that for the same period in 2019 (mean difference, −15.9%; 95% CI, −16.1% to −15.8%). Patients with pain were more likely to receive opioids (mean difference, 3.5%; 95% CI, 3.3%-3.7%) and less likely to receive nonpharmacologic therapy (mean difference, −6.0%; 95% CI, −6.3% to −5.8%), and opioid prescriptions were longer and more potent during the early pandemic in 2020 relative to 2019 (mean difference, 1.07 days; 95% CI, 1.02-1.17 days; mean difference, 0.96 morphine milligram equivalents; 95% CI, 0.76-1.20). Analysis of individuals’ transitions between treatment options for pain found that patients were more likely to transition out of nonpharmacologic therapy, replacing it with opioid prescriptions for pain management during the COVID-19 pandemic than in the year before.

**Conclusions and Relevance:**

Nonpharmacologic therapy is a benign treatment for pain often recommended instead of opioid therapy. The decrease in nonpharmacologic therapy and increase in opioid prescription during the COVID-19 pandemic found in this cross-sectional study, especially given longer days of prescription and more potent doses, may exacerbate the US opioid epidemic. These findings suggest that it is imperative to investigate the implications of limited medical access on treatment substitution, which may increase patient risk, and implement policies and guidelines to prevent those substitutions.

## Key Points

**Question:**

Was nonpharmacologic therapy (ie, physical therapy and complementary medicine)—a low-risk alternative treatment for acute and chronic pain—replaced by prescription opioid analgesics during the COVID-19 pandemic?

**Findings:**

This cross-sectional study of weekly claims data from 24 million commercially insured patients in the US found evidence of substitution of nonpharmacologic therapy with increased opioid prescribing, accompanied by more potent and longer prescriptions, at the population and individual levels during the early months of the COVID-19 pandemic.

**Meaning:**

These findings suggest that progress toward reversing the opioid epidemic may have been stalled by the pandemic as practitioners resorted to higher levels of opioid prescribing to control pain in the absence of less risky alternatives.

## Teaser

This cross-sectional study of claims data from 24 million US patients in a nationwide commercial insurance database assesses patterns of pain management treatment in the period before the COVID-19 pandemic vs during the COVID-19 pandemic.

## Introduction

Since the beginning of the COVID-19 pandemic, emergency department visits and hospital admissions associated with opioid overdose have spiked in the US.^1,2,3,4,5^ Factors such as limited access to treatment for opioid use disorders during the pandemic likely contributed to the increase of opioid use disorder diagnoses and opioid overdose deaths.^6,7,8^ In tandem, individuals’ fear of contracting COVID-19, coupled with social distancing policies that restricted elective and nonurgent medical visits,^9^ may have temporarily altered the landscape of pain management for those with chronic pain, generating renewed challenges for the US opioid epidemic.^10^

Opioids are a relatively inexpensive and effective treatment option for patients experiencing chronic pain.^11^ However, they pose a significant risk of addiction and misuse, contributing to the opioid epidemic in the US. In response to the epidemic, nonpharmacologic therapies, including physical therapies and complementary medicine, have garnered attention as safer alternative treatments for managing nonmalignant chronic pain.^12,13,14,15^ However, nonpharmacologic therapies are more expensive and time-consuming than opioid therapy. The COVID-19 pandemic added an additional barrier because these treatments require physical contact with health care professionals. Because of the risk of COVID-19 infection from close physical contact, many states enacted policies that discouraged nonurgent medical care to preserve health care capacity to cope with the COVID-19–related surge in use, and many patients elected to forgo health care visits to reduce their transmission risk.^9,16,17^ These issues raise questions about how the COVID-19 pandemic has affected patterns of pharmacologic and nonpharmacologic therapy for pain management.

Existing studies^18,19,20,21^ provide inconclusive evidence of the effect of the COVID-19 pandemic on pain management. A recent study^18^ that used a national sample of urine drug tests ordered by health care professionals found a substantial increase in the prevalence of riskier opioids, such as fentanyl and heroin, after the national emergency declaration on March, 13, 2020, although contradictory evidence has also emerged.^19,20^ Another study^21^ reported that outpatient and elective interventional procedures, including exercise therapy, massage therapy, and spinal manipulation, were significantly interrupted during the pandemic. However, because the study used a small convenience sample, it may lack external validity.

Our study aims to resolve these discrepancies by providing a comprehensive assessment of treatment use patterns for pain management, comparing the period before the COVID-19 pandemic with the period during the COVID-19 pandemic using large-scale claims data from patients representative of the commercially insured and Medicare Advantage populations in the US.^22^ We hypothesized that during the pandemic, nonpharmacologic therapy may have been replaced with prescription opioids, which do not require physical contact or even an office visit. This treatment substitution may have contributed to an increased risk of opioid-related overdoses or incident cases of substance use disorder among those with acute and chronic pain. Because of the unexpectedness of the COVID-19 pandemic, the event provides a crucial opportunity to assess the extent to which physicians and patients may rely on riskier but easier solutions (eg, opioids) vs difficult but safer ones (eg, nonpharmacologic therapy) under conditions of limited access.

## Methods

### Data Collection

This retrospective, observational cross-sectional study used weekly claims data from 24 million US patients in a nationwide commercial insurance database (Optum’s deidentified Clinformatics Data Mart Database) from January 1, 2019, to September 31, 2020. This database includes 20% of the commercially insured population in all 50 US states and Washington, DC, and 24% of the Medicare Advantage population, which was of similar age and sex to the US commercially insured population more broadly.^23,24^ Among patients in the database with diagnoses of limb, extremity, or joint pain, back pain, and neck pain for each week, patterns of treatment use were identified and evaluated. Data analysis was performed from April 1, 2021, to September 31, 2021. This study was approved by the Indiana University institutional review board and followed the Strengthening the Reporting of Observational Studies in Epidemiology (STROBE) reporting guideline. Owing to the use of deidentified patient data, the need for informed consent was waived by the institutional review board.

Using the deidentified Clinformatics Data Mart Database, a previous study^25^ reported that annual opioid use prevalence was 14% for commercial beneficiaries, 26% for aged Medicare beneficiaries, and 52% for disabled Medicare beneficiaries. Our analytic sample includes 21 430 339 who enrolled during the first 3 quarters in 2019 and 20 759 788 patients who enrolled during the first 3 quarters of 2020, excluding 3395 patients whose age and sex information are missing. The eFigure in the Supplement shows that the numbers of enrolled patients per week in 2020 during the study period decreased slightly compared with the number in 2019. The decrease in enrolled patients may be attributable to the increase in unemployment and related loss of employer-sponsored insurance or deaths due to COVID-19. To account for the changing population, we used the number of enrollees in a week as the denominator when we calculated the weekly rate of patients with a pain diagnosis.

### Diagnosis of Pain

For this study, we relied on the *International Statistical Classification of Diseases, Tenth Revision, Clinical Modification (ICD-10-CM)* code list that includes a full spectrum of common pain conditions.^26^ We used a subset of 10 548 *ICD-10-CM* codes to identify patients with any of these common pain conditions: limb or extremity pain; joint pain and nonsystemic, noninflammatory arthritic disorders; back pain; and neck pain. eTable 1 in the Supplement lists the numbers of patients who received each pain diagnosis cluster in 2019 and 2020.

### Opioid and Nonpharmacologic Therapy for Pain Treatment

We designated patients as at risk if they received any pain diagnosis in the week regardless of previous pain diagnoses. Of patients with pain, 2 types of treatments were measured. First, we identified prescriptions for opioid analgesics using the list of National Drug Codes provided by the National Center for Injury Prevention and Control and created a binary indicator for whether a patient received any opioid prescription each week. eTable 2 in the Supplement lists the numbers of patients with pain receiving different opioid drugs; acetaminophen-hydrocodone and tramadol hydrochloride were most frequently used, followed by acetaminophen–oxycodone hydrochloride and oxycodone hydrochloride.

Second, to examine the duration and strength of opioid prescriptions, for each week we measured days of opioid prescriptions and total morphine milligram equivalents (MMEs). We calculated the length in days of the opioid prescription using the maximum length of days for which each drug is prescribed and taking the sum of all the days prescribed in a given week for a patient. We calculated MMEs for each opioid prescription using strength per unit × (number of units/days’ supply) × MME conversion factor^12^ and took the sum of MMEs for all prescriptions received by a patient in a given week. In addition, we identified the nonpharmacologic treatment for pain using 21 *Current Procedural Terminology* codes (eTable 3 in the Supplement) and created a binary indicator of whether a patient received any of these nonpharmacologic therapies in a given week. eTable 4 in the Supplement lists the numbers of patients with pains receiving different types of nonpharmacologic therapy for pain treatment.

### Statistical Analysis

We described weekly trends in pain prevalence from the first week to the 40th week of 2020 using trends from the same period in 2019 as a baseline for comparison. We used the same strategy to describe weekly trends in indicators of opioid prescriptions and nonpharmacologic therapy for patients with pain. In doing so, we calculated the proportion of patients with pain in a given week and the prevalence of treatment use (ie, opioids and nonpharmacologic therapy) as well as the length in days and strength in MMEs of opioid prescriptions among patients with pain who visited physicians in the same week.

To conduct statistical tests for changes in these indicators from 2019 to 2020, we broadly defined the prepandemic (weeks 1-10; before the national emergency declaration on March 13, 2020), the early pandemic (weeks 11-27; until Independence Day, encompassing state closures followed by partial reopening with capacity constraints and service backlogs), and the later pandemic period (weeks 28-40) for 2020 based on 2 major discontinuities in the COVID-19 pandemic in the US.^7,27^ We compared differences in the proportion of patients with pain, patients with pain receiving opioids, and patients with pain receiving nonpharmacologic therapy, as well as the mean of weekly total MMEs and the mean of weekly total days of opioid prescriptions between 2019 and 2020 during the prepandemic and the early pandemic period, respectively, using linear probability models for binary outcomes and regression models for continuous outcomes with robust SEs that account for clustering across overlapping patients between 2019 and 2020. Specifically, we regressed the outcome for each patient within each period on an indicator for the pandemic year (taking a value of 1 in 2020 and 0 in 2019) and interpreted the coefficient on the 2020 indicator as the difference in mean outcomes between 2019 and 2020. We present 95% CIs to characterize the uncertainty associated with our estimates of the trends and differences.

Finally, we investigated how patients transitioned between treatment options by producing a Markov transition matrix. We assigned patients to 4 different categories based on the treatment they received each week. Specifically, a patient might receive no treatment, only opioids, only nonpharmacologic therapy, or both opioid and nonpharmacologic therapy simultaneously in a given week. We only included patients who visited at least in 2 weeks in 2019 and 2020 so that we could identify the transition in treatment options between 2 consecutive visits. We then calculated the rates at which patients transitioned from one category to another in consecutive weeks and aggregated these rates for the prepandemic and early pandemic periods. We produced the same transition matrix for the corresponding 2 periods in 2019 as a baseline. To compare changes between 2019 and 2020 for the same period, we calculated mean differences in the transition rates between 2019 and 2020 during the prepandemic and early pandemic periods, respectively.

Statistical analyses were performed on September 5, 2021, using R statistical software, version 4.0.3 (R Foundations for Statistical Computing) and Stata MP, version 17 (StataCorp LLC). All codes and data to replicate analyses are available on the Harvard dataverse website.^28^

## Results

A total of 21 430 339 patients (mean [SD] age, 48.6 [24.0] years; 10 960 507 [51.1%] female; 909 061 [4.2%] Asian, 1 688 690 [7.9%] Black, 2 276 075 [10.6%] Hispanic, 11 192 789 [52.2%] White, and 5 363 724 [25.0%] unknown) were enrolled during the first 3 quarters in 2019 and 20 759 788 (mean [SD] age, 47.0 [23.8] years; 10 695 690 [51.5%] female; 798 037 [3.8%] Asian; 1 508 023 [7.3%] Black, 1 976 248 [9.5%] Hispanic, 10 059 597 [48.5%] White, and 6 417 883 [30.9%] unknown) in the first 3 quarters of 2020. eTable 5 in the Supplement summarizes the characteristics of all enrolled patients and patients with pain in 2019 and 2020. Among 20 759 788 enrollees in 2020, 5 280 231 patients (25.4%; 3 046 909 [57.7%] female) received at least 1 pain diagnosis vs 5 834 947 (27.2%) of 21 430 339 enrollees (3 357 288 [57.5%] female) observed in 2019. The characteristics of the patients with pain in 2020, including pain type, were similar to those in 2019, except that patients with pain in 2020 were more likely to be privately insured and their race and ethnicity were less likely to be unknown.

**Figure 1.**
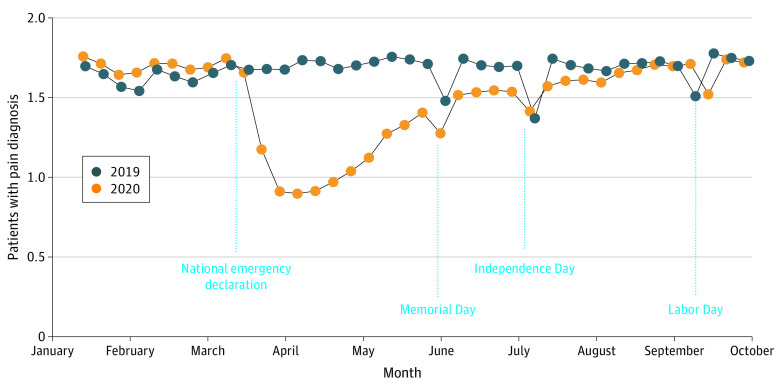
Trends in the Share of Patients With Selected Pain Diagnoses in 2019 and 2020 The proportion of patients receiving a pain diagnosis is calculated as the ratio of the number of patients with pain to the number of patients with any claim in a week in 2019 and 2020. There were decreases around 3 major holidays as well as the national emergency declaration on March 13, 2020.

Figure 1 shows that the proportion of patients who received any pain diagnosis in 2020 was similar to that from 2019 until March but decreased starting the third week of March 2020, right after the national emergency declaration. The Table indicates that the decrease in the proportion of patients with pain in the early pandemic period from 2019 to 2020 was substantial (mean difference, −15.9%; 95% CI, −16.1% to −15.8%), given that the proportion of patients with pain slightly increased from 2019 to 2020 in the prepandemic period (mean difference, 0.4%; 95% CI, 0.4%-0.5%). The prevalence of pain diagnoses was lower until August 2020 but rebounded to the 2019 level in September. To account for the influence of limited medical access, we restricted our analysis to patients who received a pain diagnosis each week.

**Table.** Changes in the Share of Patients With Pain and Patterns of Pain Treatment From 2019 to 2020 Across 3 COVID-19 Pandemic Phases^a^

Variable	Mean (95% CI)								
	Prepandemic period			Early pandemic period			Later pandemic period		
	2019	2020	Difference	2019	2020	Difference	2019	2020	Difference
Patients with pain, %	31.6 (31.6 to 31.7)	32.1 (32 to 32.1)	0.4 (0.4 to 0.5)	59.5 (59.4 to 59.6)	43.6 (43.5 to 43.6)	−15.9 (−16 to −15.8)	44.3 (44.3 to 44.4)	44.7 (44.7 to 44.8)	0.4 (0.3 to 0.5)
Patients with pain receiving any nonpharmacologic therapy, %	35.2 (35.1 to 35.4)	35.8 (35.6 to 36)	0.6 (0.4 to 0.8)	45.9 (45.7 to 46.1)	39.8 (39.6 to 40.0)	−6.0 (−6.3 to −5.8)	39.6 (39.4 to 39.8)	37.5 (37.3 to 37.7)	−2.1 (−2.4 to −1.9)
Patients with pain receiving any opioid, %	25.5 (25.4 to 25.7)	24.2 (24.1 to 24.3)	−1.4 (−1.5 to −1.2)	28.6 (28.5 to 28.8)	32.1 (31.9 to 32.3)	3.5 (3.3 to 3.7)	25.7 (25.6 to 25.8)	26.7 (26.5 to 26.8)	0.9 (0.8 to 1.1)
Sum of total									
Days of opioid prescription	5.5 (5.5 to 5.5)	5.2 (5.2 to 5.2)	−0.28 (−0.32 to −0.20)	5.8 (5.7 to 5.8)	6.8 (6.8 to 6.9)	1.07 (1.02 to 1.1)	5.4 (5.4 to 5.5)	5.7 (5.6 to 5.7)	0.23 (0.19 to 0.30)
MMEs of opioid prescription	12.1 (11.9 to 12.2)	10.7 (10.6 to 10.8)	−1.37 (−1.52 to −1.20)	12.8 (12.6 to 12.9)	13.7 (13.6 to 13.9)	0.96 (0.76 to 1.20)	11.5 (11.4 to 11.6)	11.5 (11.4 to 11.6)	−0.02 (−0.18 to 0.10)

Abbreviation: MMEs, morphine milligram equivalents.

^a^ This table presents the mean (95% CI) of each outcome in 2019 and 2020 across the prepandemic (weeks 1-10), early pandemic (weeks 11-27), and later pandemic (weeks 28-40) periods. The changes from 2019 to 2020 are presented with 95% CIs estimated from regression models that account for clustering because of overlapping patients across different periods.

**Figure 2.**
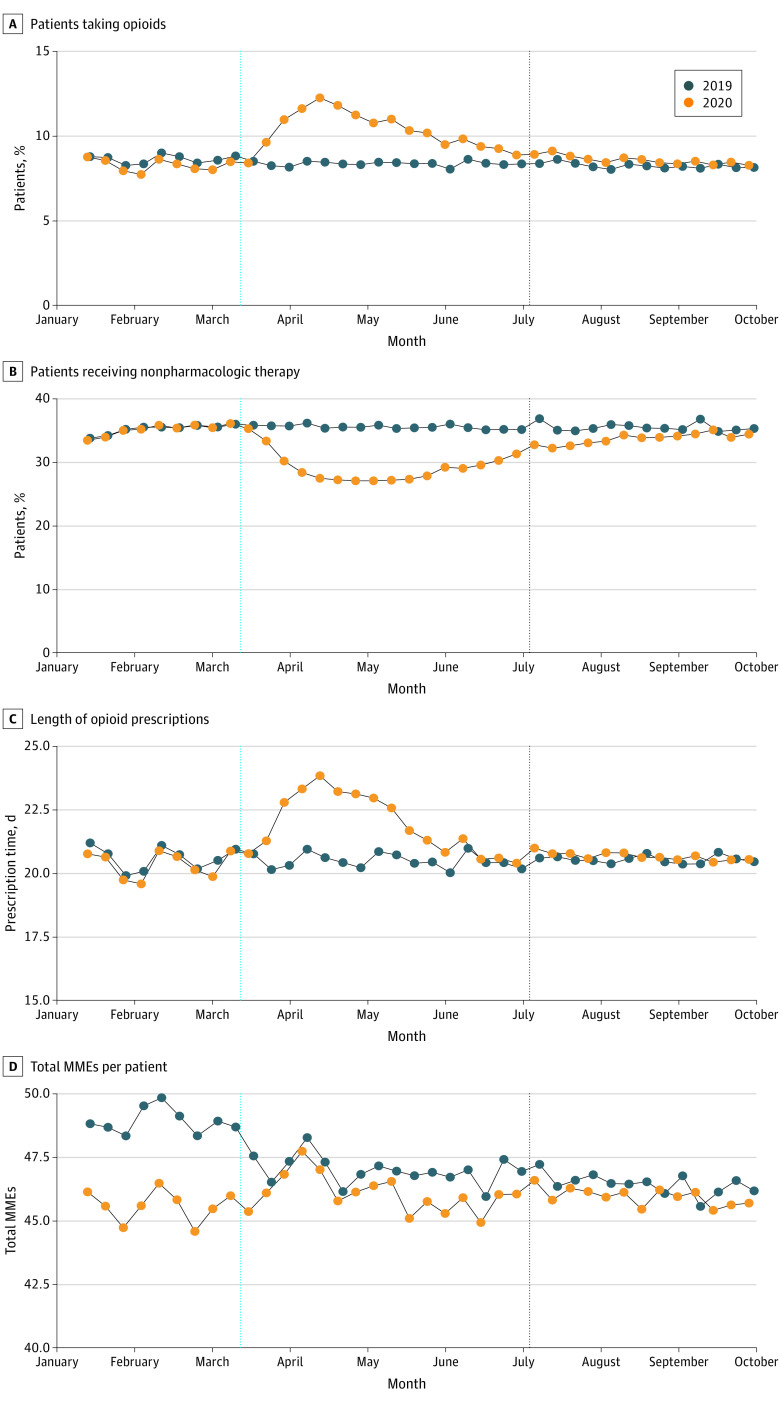
Weekly Trends in the Share of Patients With Pain and the Patterns of Pain Treatment A and B, Patients could have received both opioids and nonpharmacologic therapy. Blue vertical lines indicate the date of the national emergency declaration (March 13, 2020), and black dotted lines indicate the date of Independence Day (July 4, 2020). The extremely narrow 95% CIs are not visible because of the large sample size and indicate very precise estimates. MMEs indicates morphine milligram equivalents.

Figure 2A and B shows how patients with pain who could have received both opioids and nonpharmacologic therapy were treated during the COVID-19 pandemic. The proportion of patients with pain receiving opioids decreased in the prepandemic period in 2020 compared with 2019 (−1.4%; 95% CI, −1.5% to −1.2%) but increased in the early pandemic period in 2020 compared with 2019 (3.5%; 95% CI, 3.3%-3.7%). In addition, the proportion of patients with pain receiving nonpharmacologic therapy was lower in the early pandemic period in 2020 compared with 2019 (−6.0%; 95% CI, −6.3% to −5.8%), which contradicts the prepandemic trends (0.6%; 95% CI, 0.4% to 0.8%). Patients with pain were also more likely to receive longer opioid prescriptions in the early pandemic in 2020 than in 2019 (mean difference, 1.07 days; 95% CI, 1.02-1.17 days), although this deviation quickly returned to the 2019 level in the later pandemic period (mean difference, 0.23 days; 95% CI, 0.19-0.30 days). Patients with pain received opioid prescriptions with higher doses in the early pandemic in 2020 than in 2019 (mean difference, 0.96 MMEs; 95% CI, 0.76-1.20 MMEs), although the dosage level decreased in the prepandemic period (mean difference, −1.37 MMEs; 95% CI, −1.52 to −1.2 MMEs) and decreased again in the later pandemic period. The observed changes in opioid and nonpharmacologic therapy for pain remain broadly similar for alternative definitions of the prepandemic, early pandemic, and later pandemic periods in 2020, exhibiting a decrease during state closures and early reopenings followed by subsequent recovery to 2019 levels.

**Figure 3.**
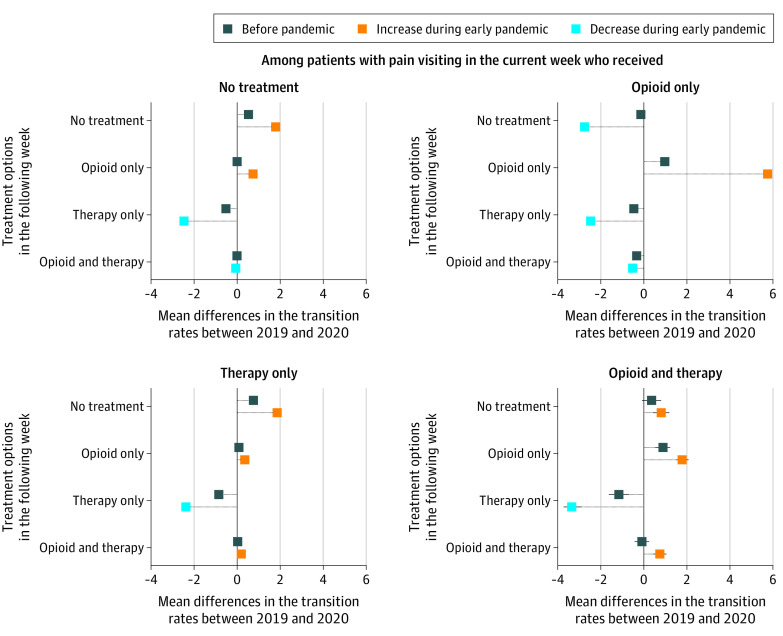
Comparison of Transition Rates Across Opioid and Nonpharmacologic Therapy for Patients With Pain in 2019 and 2020 Transition rate matrixes were generated for the prepandemic (weeks 1-10) and the early pandemic (weeks 11-27) periods using weekly claims and prescription data by calculating whether each patient received no treatment, opioids only, nonpharmacologic therapy only, or both opioids and therapy in the next visit conditional on the treatment received in the current visit. Each square shows the difference in transition rates from 2019 to 2020 during the prepandemic period (green) and the early pandemic period (orange for increases; blue for decreases). See eTables 6 and 7 in the Supplement for prepandemic and early pandemic transition matrixes for 2019 and 2020 and eTable 8 in the Supplement for the difference in transition rates from 2019 to 2020.

The patient-level transition rates across the 4 different treatment options (ie, received no treatment, opioid therapy only, nonpharmacologic therapy only, and both opioid and nonpharmacologic therapy) during the prepandemic and the early pandemic period in 2019 and 2020 are presented in eTables 6 and 7 in the Supplement, respectively. Figure 3 shows the mean differences in transition rates among patients with pain from 2019 to 2020 in the prepandemic and early pandemic periods. In all cases, the transition to nonpharmacologic therapy decreased in the early pandemic period in 2020, whereas the transition to opioids increased compared with the 2019 baseline. However, the prepandemic differences in transition rates across different treatment options from 2019 to 2020 were very small compared with those in the early pandemic period.

eTable 8 in the Supplement indicates that for the early pandemic period in 2020, compared with 2019, those who received no treatment during the current visit were more likely to receive only opioids during the next visit (mean difference, 0.74%; 95% CI, 0.71%-0.78%) and were less likely to receive nonpharmacologic therapy only (mean difference, −2.47%; 95% CI, −2.51% to −2.42%). Those patients who received only opioids during the current visit were more likely to receive opioids in the next visit (mean difference, 5.75%; 95% CI, 5.56%-5.94%) and were less likely to receive nonpharmacologic therapy (mean difference, −2.47%, 95% CI, −2.57% to −2.37%). Those who received only nonpharmacologic therapy in the current visit were more likely to receive opioids only (mean difference, 0.36%; 95% CI, 0.33%-0.38%), both opioids and nonpharmacologic therapy (mean difference, 0.17%; 95% CI, 0.14%-0.19%), or no treatment (mean difference, 1.86%; 95% CI, 1.78%-1.94%) in the next visit and were less likely to receive nonpharmacologic therapy (mean difference, −2.38%; 95% CI, −2.47%- −2.29%). Those who received both opioids and nonpharmacologic therapy during the current visit were more likely to receive opioids only (mean difference, 1.78%; 95% CI, 1.51%-2.05%) or no treatment (mean difference, 0.81%; 95% CI, 0.43%-1.18%) and were less likely to receive nonpharmacologic therapy only (mean difference, −3.32%; 95% CI, −3.73% to −2.92%).

## Discussion

In this cross-sectional study, we examined the treatment of acute and chronic pain during the early period of the COVID-19 pandemic. With the exponential spread of COVID-19 and the resulting US national emergency declaration, in-person health care visits for elective, nonessential medical care were suspended by many states, practitioners, and patients to reduce community transmission. Using population-level trends and individual-level treatment transitions, we assessed the consequences of these circumstances for pain treatment.

Although the adaptive prescribing response during the shutdowns in March through June 2020 may have provided a necessary stopgap for patients in need of pain management, it may also have increased patient risk. The significant decrease in the proportion of patients with pain during the early COVID-19 pandemic period identified in our study implies that many patients may have experienced pain without an effective treatment because they could not visit physicians and hospitals because of COVID-related restrictions and concerns. Even among patients who could obtain health services, patients with pain using nonpharmacologic therapy alone or in combination with opioid maintenance therapy may have had to introduce opioids or increase their dosage, respectively, to manage pain during a period of limited access to nonpharmacologic therapy. Likewise, patients newly diagnosed with acute pain may have been introduced to opioids when an equally effective nonpharmacologic therapy would typically have been prescribed. Excessive pandemic-driven exposures to opioids and to higher doses and longer prescriptions may have increased the risk of future misuse or dependence.^29,30,31,32^

In 2016, the Centers for Disease Control and Prevention recommended use of nonpharmacologic therapy and nonopioid pharmacologic therapy instead of or in combination with opioid therapy because those options effectively reduce pain and improve physical function without the risk of addiction.^12,13^ Because these recommendations alone have not had significant effects on the opioid epidemic, policies that target the opioid epidemic have continued to focus on managing and controlling the supply of prescription opioids.^33^ Recent studies^24,34,35^ have found that the success of these policies—shorter duration and lower volume of opioid prescriptions—is not sufficient to reverse the increasing trends in opioid overdose mortality. Against this background, our findings regarding the substitution of nonpharmacologic therapy with opioid therapy during the COVID-19 pandemic suggest that policies that markedly expand the use of nonaddictive treatments, such as physical therapy for chronic pain management, are urgently needed.

Although the proliferation of telemedicine during the COVID-19 pandemic^36^ may have contributed to the substitution of nonpharmacologic therapy with opioid prescriptions, virtual nonpharmacologic therapy holds potential to help reduce disparities caused by unequal access to in-person nonpharmacologic therapy. Although certain procedures require direct in-person contact (eg, deep tissue massage), other forms of therapy can be effectively provided remotely through telemedicine (eg, certain physical therapies).^37^ Although the digital divide remains a problem, especially in rural areas and among patients with low socioeconomic status,^38,39^ telemedicine could be a viable strategy to provide nonpharmacologic therapy to patients with pain under social distancing or to those in areas with a shortage of health care professionals.

These findings on pain management should be considered in the context of pandemic-associated changes in the opioid epidemic. Recent estimates from the Centers for Disease Control and Prevention suggest that more than 81 000 drug overdose deaths occurred in the US in the 12 months ending in May 2020.^2^ This estimate is the highest number of overdose deaths ever recorded in a 12-month period. This recent increase in overdose mortality is a sharp deviation from trends before the pandemic. Specifically, during 2017 to 2018, opioid-involved overdose deaths had decreased for the first time since 1999. Although synthetic opioids appear to be driving the increase in overdose deaths, increasing 38.4% from the prior year, our findings on excessive exposure to prescription opioids during the pandemic may portend future problems, potentially disrupting large-scale public health efforts to reduce inappropriate prescribing and encourage nonpharmacologic therapies for pain.

### Limitations

Our study has limitations. First, because the data used for the analysis are only nationally representative of the commercially insured and Medicare Advantage populations, our results may not generalize to other populations (eg, those who are uninsured or covered by Medicaid). This issue may be particularly problematic during the COVID-19 pandemic because high rates of unemployment could have led to reduced commercial coverage, although our data indicate only a marginal decrease in the enrolled population. Second, because our data reflect only insurance claims, any medications or treatments paid for directly or obtained outside the insurance system would not be captured in our data set, which could bias our results. Third, our measure of opioid dosage per week is based on whether the patient received a prescription on the claim and does not reflect whether the person actually consumed the medication. Fourth, although our findings regarding longer and higher-dosage opioid prescriptions in the early months of the pandemic raise concerns about the downstream harms associated with the deviations from recommended opioid prescribing patterns, the examination of possible long-term effects is left to future work as additional data become available.

## Conclusions

Projecting beyond the pandemic, our findings on substitution of nonpharmacologic therapy with opioids may have broader implications for health disparities.^40^ We found that under conditions of reduced access to diverse treatment options, practitioners and patients resorted to riskier alternatives to manage acute and chronic pain. After the pandemic, nonpharmacologic therapy will likely continue to be inaccessible for many patients because of factors such as cost, underinsurance, lack of transportation, lack of childcare, or inability to take time off work. These barriers disproportionately affect people in rural areas, Black and Latinx patients, gender and sex minorities, and those in disadvantaged socioeconomic groups^41,42,43^ and thus may contribute to broader disparities in opioid use disorders and overdose. It is critical to increase universal access to nonpharmacologic treatments for pain management by reducing these barriers.

## Notes

Supplement. **eFigure.** Weekly Trends of Patient Enrollment From the First Quarter to the Third Quarter in 2019 and 2020 **eTable 1.** Number of Patients Who Receive Pain Diagnoses Across Three Pandemic Phases in 2019 and 2020 **eTable 2.** Number of Pain Patients Receiving Opioid Drugs Across Three Pandemic Phases in 2019 and 2020 **eTable 3.** List of CPT Codes for Nonpharmacologic Therapy **eTable 4.** Number of Pain Patients Receiving Nonpharmacologic Therapy for Three Pandemic Phases in 2019 and 2020 **eTable 5.** Characteristics of the Study Sample From the First Quarter to the Third Quarter in 2019 and 2020 **eTable 6.** Markov Transition Matrices From the Current Treatment Options to the Following Treatment Options Among Pain Patients in 2019 **eTable 7.** Markov Transition Matrices From the Current Treatment Options to the Following Treatment Options Among Pain Patients in 2020 **eTable 8.** Mean Differences in Transition Rates From the Current Treatment Options to the Following Treatment Options Among Pain Patients From 2019 to 2020
