# Error in a Figure

**DOI:** 10.1001/jamanetworkopen.2021.46308

**Published:** 2022-01-07

In the Original Investigation titled “Substitution of Nonpharmacologic Therapy With Opioid Prescribing for Pain During the COVID-19 Pandemic,”^1^ published December 10, 2021, Figure 1 has been amended to show the trends in the share of patients with any pain diagnosis in 2019 and 2020. The graph that was published originally depicted trends in the share of patients with back pain. This change does not affect the authors’ conclusions. This article has been corrected.
