# Supplementary material for: Substitution of Nonpharmacologic Therapy With Opioid Prescribing for Pain During the COVID-19 Pandemic
Source: JAMA Netw Open. 2021 Dec 10;4(12):e2138453. doi: 10.1001/jamanetworkopen.2021.38453 (PMC8665369; doi:10.1001/jamanetworkopen.2021.38453)
Supplement: Supplement. — eFigure. Weekly Trends of Patient Enrollment From the First Quarter to the Third Quarter in 2019 and 2020 eTable 1. Number of Patients Who Receive Pain Diagnoses Across Three Pandemic Phases in 2019 and 2020 eTable 2. Number of Pain Patients Receiving Opioid Drugs Across Three Pandemic Phases in 2019 and 2020 eTable 3. List of CPT Codes for Nonpharmacologic Therapy eTable 4. Number of Pain Patients Receiving Nonpharmacologic Therapy for Three Pandemic Phases in 2019 and 2020 eTable 5. Characteristics of the Study Sample From the First Quarter to the Third Quarter in 2019 and 2020 eTable 6. Markov Transition Matrices From the Current Treatment Options to the Following Treatment Options Among Pain Patients in 2019 eTable 7. Markov Transition Matrices From the Current Treatment Options to the Following Treatment Options Among Pain Patients in 2020 eTable 8. Mean Differences in Transition Rates From the Current Treatment Options to the Following Treatment Options Among Pain Patients From 2019 to 2020 [file jamanetwopen-e2138453-s001.pdf]

Supplementary Online Content

Lee B, Yang KC, Kaminski P, et al. Substitution of nonpharmacologic therapy with opioid prescribing for pain during the COVID-19 pandemic. *JAMA Netw Open*. 2021;4(12):e2138453. doi:10.1001/jamanetworkopen.2021.38453

eFigure. Weekly Trends of Patient Enrollment From the First Quarter to the Third Quarter in 2019 and 2020

eTable 1. Number of Patients Who Receive Pain Diagnoses Across Three Pandemic Phases in 2019 and 2020

eTable 2. Number of Pain Patients Receiving Opioid Drugs Across Three Pandemic Phases in 2019 and 2020

eTable 3. List of CPT Codes for Nonpharmacologic Therapy

eTable 4. Number of Pain Patients Receiving Nonpharmacologic Therapy for Three Pandemic Phases in 2019 and 2020

eTable 5. Characteristics of the Study Sample From the First Quarter to the Third Quarter in 2019 and 2020

eTable 6. Markov Transition Matrices From the Current Treatment Options to the Following Treatment Options Among Pain Patients in 2019

eTable 7. Markov Transition Matrices From the Current Treatment Options to the Following Treatment Options Among Pain Patients in 2020

eTable 8. Mean Differences in Transition Rates From the Current Treatment Options to the Following Treatment Options Among Pain Patients From 2019 to 2020

This supplementary material has been provided by the authors to give readers additional information about their work.

eFigure. Weekly Trends of Patient Enrollment From the First Quarter to the Third Quarter in 2019 and 2020

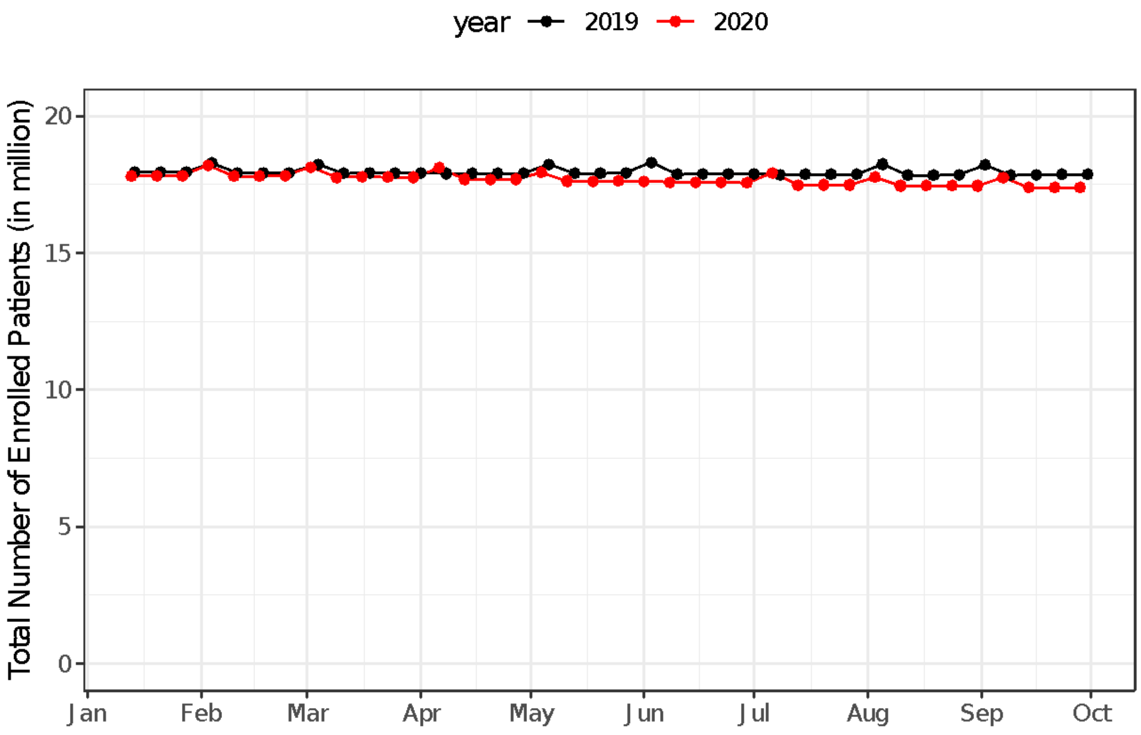

© 2021 Lee B et al. *JAMA Network Open.*

eTable 1. Number of Patients Who Receive Pain Diagnoses Across Three Pandemic Phases in 2019 and 2020

Year / Period<br>Diagnosis Cluster	2019<br>Pre	2020<br>Pre	2019<br>Early	2020<br>Early	2019<br>Later	2020<br>Later
Limb and joint pain[a)]	1,936,902	1,973,647	2,825,247	2,177,338	2,334,530	2,162,256
Back pain	1,185,323	1,192,530	1,654,683	1,326,449	1,389,452	1,297,174
Neck pain	557,737	562,567	792,807	610,371	658,312	596,776

Note. In this table, we report the number of patients with different pain diagnoses for three pandemic phases in 2019 and 2020. Due to limited space, we aggregate pain diagnosis by diagnosis cluster (see Mayhew et al 2019 for the full list). Pre: Pre-pandemic Period (weeks 1-10), Early: Early pandemic Period (weeks 11-27), Later: Later pandemic Period (weeks 28-40).
[a)] Limb and joint pain: Limb/extremity pain, joint pain and non-systemic, non-inflammatory arthritic disorders

© 2021 Lee B et al. *JAMA Network Open.*

eTable 2. Number of Pain Patients Receiving Opioid Drugs Across Three Pandemic Phases in 2019 and 2020

Year / Period<br>Generic Drug Name	2019<br>Pre	2020<br>Pre	2019<br>Early	2020<br>Early	2019<br>Later	2020<br>Later
Acetaminophen/Hydrocodone Bitartrate	145043	208511	161810	138959	181129	157075
Tramadol Hydrochloride	97991	156777	120126	95958	129782	114889
Acetaminophen/Oxycodone Hydrochloride	68116	100057	77061	65996	80940	74203
Oxycodone Hydrochloride	50194	70222	57965	53271	63168	62113
Acetaminophen/Codeine Phosphate	25586	42515	31497	24136	31356	27225
Morphine Sulfate	16715	21208	17957	15937	19462	17354
Hydromorphone Hydrochloride	6534	9233	7332	6394	7737	7691
Fentanyl	5179	6694	5036	3775	4684	4234
Methadone Hydrochloride	3738	4168	3097	2841	4210	3895
Acetaminophen/Tramadol Hydrochloride	3179	5191	3942	2278	2883	2325
Oxycodone	3055	4186	3645	3548	4373	3948
Buprenorphine/Naloxone	2426	2736	1876	1426	1790	1591
Buprenorphine Hydrochloride	1083	1801	1464	1009	1353	1260
Hydrocodone Bitartrate	825	1109	883	773	985	800
Hydrocodone Bitartrate/Ibuprofen	710	966	709	520	609	486
Morphine Sulfate/Naltrexone Hydrochloride	639	825	703	91	8	
Oxymorphone Hydrochloride	484	579	398	87	93	86
Buprenorphine	280	325	242	184	202	148
Apap/Butalbital/Caff/Codeine Phos	246	345	299	234	297	261
Butorphanol Tartrate	174	237	200	157	225	183
Meperidine Hydrochloride	160	224	99	31	17	11
Aspirin/Butalbital/Caffeine/Codeine Phosphate	152	255	172	121	108	81
Acetaminophen/Caffeine/Dihydrocodeine Bitartrate	129	223	190	105	125	126
Levorphanol Tartrate	103	90	59	41	27	22
Naloxone Hydrochloride/Pentazocine Hydrochloride	102	164	115	87	101	101
Codeine Sulfate	84	136	86	64	79	72
Aspirin/Oxycodone Hydrochloride	24	27	22	4	5	2
Tapentadol Hydrochloride	23					
Fentanyl Citrate	14	20	18	12	8	7
Belladonna Alkaloids/Opium Alkaloids	12	16	7	6	4	5
Propoxyphene Hydrochloride	3			1		
Aspirin/Carisoprodol/Codeine Phosphate	2					
Ibuprofen/Oxycodone Hydrochloride		3	2	1	1	1

Note. In this table, we report the number of patients with different opioid prescriptions for three pandemic phases in 2019 and 2020. Due to limited space, we aggregate the opioid drugs by their generic names. Pre: Pre-pandemic Period (weeks 1-10), Early: Early pandemic Period (weeks 11-27), Later: Later pandemic Period (weeks 28-40).

© 2021 Lee B et al. *JAMA Network Open.*

eTable 3. List of CPT Codes for Nonpharmacologic Therapy

CPT code	Procedure
64550	Application of surface transcutaneous neurostimulator (discontinued in CPT 2019)
97014	Supervised electrical stimulation (unattended)
97032	Electrical stimulation (constant attendance)
97110	Therapeutic exercise
97112	Neuromuscular re-education
97113	Aquatic therapy with therapeutic exercises
97116	Gait training
97124	Massage therapy
97127	Therapeutic interventions that focus on cognitive function (effective Jan 1, 2018)
97140	Manual therapy
97150	Therapeutic procedure(s), group (2 or more individuals)
97530	Therapeutic activities, direct (one-on-one) patient contact
97532	Development of cognitive skills (replaced code 97127 effective Jan 1, 2018)
97810	Acupuncture without electrical stimulation
97811	Acupuncture without electrical stimulation (additional 15 minutes)
97813	Acupuncture with electrical stimulation
97814	Acupuncture with electrical stimulation (additional 15-minutes)
98940	Chiropractic manipulative treatment (CMT); spinal, 1-2 regions
98941	Chiropractic manipulative treatment (CMT); spinal, 3-4 regions
98942	Chiropractic manipulative treatment (CMT); spinal, 5 regions
98943	Chiropractic manipulative treatment (CMT); extraspinal, 1 or more regions

© 2021 Lee B et al. *JAMA Network Open.*

eTable 4. Number of Pain Patients Receiving Nonpharmacologic Therapy for Three Pandemic Phases in 2019 and 2020

Year / Period<br>Procedure	2019<br>Pre	2020<br>Pre	2019<br>Early	2020<br>Early	2019<br>Later	2020<br>Later
Chiropractic Massage	828282	1236972	1008888	844569	854546	873457
Physical therapy	450560	667371	548785	460528	442735	471431
Transcutaneous electrical nerve stimulation (TENS)	46484	67950	51479	38364	38274	38109
Acupuncture	22055	30486	25524	19386	13408	15325
Massage therapy	7763	11171	8570	6519	5940	5871
Cognitive behavioral therapy	125	206	132		1	

Note: We used the list of CPT codes shown in eTable 3 to capture non-opioid pain therapies. Pre: Pre-pandemic Period (weeks 1-10), Early: Early pandemic Period (weeks 11-27), Later: Later pandemic Period (weeks 28-40).

© 2021 Lee B et al. *JAMA Network Open.*

eTable 5. Characteristics of the Study Sample From the First Quarter to the Third Quarter in 2019 and 2020

	All patients		Pain patients	
Year	2019	2020	2019	2020
N enrolled patients	21,430,339	20,759,788	5,280,231	5,834,947
N visit weeks	31.7	32.1	4.3	4.1
Age	47 (23.8)	48.6 (24)	61.5 (19.3)	60 (20)
Female	10,960,507 (51.1)	10,6+95,690 (51.5)	3,046,909 (57.7)	3,357,288 (57.5)
Race				
White	11,192,789 (52.2)	10,059,597 (48.5)	2,825,327 (53.5)	3,331,385 (57.1)
Black	1,688,690 (7.9)	1,508,023 (7.3)	445,354 (8.4)	524,131 (9)
Hispanic	2,276,075 (10.6)	1,976,248 (9.5)	442,610 (8.4)	540,823 (9.3)
Asian	909,061 (4.2)	798,037 (3.8)	133,258 (2.5)	174,428 (3)
Unknown	5,363,724 (25)	6,417,883 (30.9)	1,433,682 (27.2)	1,264,180 (21.7)
Insurance type[a)]				
POS	11,053,800 (51.6)	10,202,387 (49.1)	1,696,485 (32.1)	2,050,094 (35.1)
OTH	4,215,290 (19.7)	4,813,216 (23.2)	2,165,258 (41)	2,142,205 (36.7)
HMO	3,683,804 (17.2)	3,530,287 (17)	926,768 (17.6)	1,046,751 (17.9)
EPO	1,836,693 (8.6)	1,718,857 (8.3)	275,321 (5.2)	326,417 (5.6)
PPO	998,248 (4.7)	873,245 (4.2)	216,145 (4.1)	265,777 (4.6)
IND	55,632 (0.3)	51,381 (0.2)	24,900 (0.5)	29,305 (0.5)
Medicare type				
MAPD Dual	576,307 (2.7)	607,943 (2.9)	301,679 (5.7)	315,298 (5.4)
MAPD LIS	683,709 (3.2)	826,113 (4)	416,837 (7.9)	393,098 (6.7)
MAPD Other	4,361,800 (20.4)	5,063,246 (24.4)	2,200,337 (41.7)	2,219,938 (38)
Privately insured	15,149,556 (70.7)	13,931,687 (67.1)	2,294,060 (43.4)	2,789,102 (47.8)
Pain diagnosis				
All pain	5,834,947 (27.2)	5,280,231 (25.4)	5,280,231 (100)	5,834,947 (100)
Limb/Joint[b)]	4,541,525 (21.2)	4,076,958 (19.6)	4,076,958 (77.2)	4,541,525 (77.8)
Back	2,654,079 (12.4)	2,394,442 (11.5)	2,394,442 (45.3)	2,654,079 (45.5)
Neck	1,328,072 (6.2)	1,170,956 (5.6)	1,170,956 (22.2)	1,328,072 (22.8)

NOTE. This table reports the number of patients or mean (% or standard deviation) for variables among all patients enrolled from January 1 to September 30, 2019, and January 1 to September 30, 2020. Some patients may be enrolled in both periods, while some are only enrolled in one of the periods. We report the statistics from the two periods separately. Note that a small number of patients have multiple insurance types in each period, and we randomly select one type for them.

[a)] POS: point of service, OTH: other, HMO: health maintenance organization, EPO: exclusive provider organization, PPO: preferred provider organization, IND: indemnity.

[b)] Limb/Joint: Limb/extremity pain, joint pain and non-systemic, non-inflammatory arthritic disorders.

© 2021 Lee B et al. *JAMA Network Open.*

eTable 6. Markov Transition Matrices From the Current Treatment Options to the Following Treatment Options Among Pain Patients in 2019

Panel A. During the pre-pandemic period.

	In the following visit				
Current visit	No treatment	Opioid only	Therapy only	Both	N
No treatment	79.2 [79.15, 79.24]	7.04 [7.01, 7.07]	12.6 [12.56, 12.64]	1.17 [1.15, 1.18]	2,956,560
Opioid only	51.15 [50.99, 51.31]	35.64 [35.49, 35.79]	9.85 [9.76, 9.95]	3.36 [3.3, 3.41]	391,234
Therapy only	17.99 [17.94, 18.05]	1.61 [1.59, 1.62]	78.49 [78.43, 78.55]	1.91 [1.89, 1.93]	1,875,563
Both	32.04 [31.74, 32.34]	13.43 [13.21, 13.65]	42.66 [42.34, 42.97]	11.87 [11.67, 12.08]	93,748
N	2,909,194	390,160	1,923,165	94,586	5,317,105

Panel B. During the early pandemic period.

	In the following visit				
Current visit	No treatment	Opioid only	Therapy only	Both	N
No treatment	79.23 [79.2, 79.27]	6.92 [6.9, 6.95]	12.69 [12.66, 12.72]	1.15 [1.14, 1.16]	4,886,869
Opioid only	51.08 [50.96, 51.2]	35.63 [35.51, 35.74]	9.95 [9.88, 10.02]	3.34 [3.3, 3.39]	645,712
Therapy only	17.44 [17.4, 17.49]	1.57 [1.55, 1.58]	79.07 [79.03, 79.11]	1.92 [1.9, 1.93]	3,280,430
Both	31.34 [31.11, 31.56]	13.44 [13.27, 13.6]	43.18 [42.94, 43.42]	12.05 [11.89, 12.21]	164,925
N	4,825,859	641,965	3,349,412	160,700	8,977,936

Note. Among pain patients who visit more than once, we measure the transition rate using weekly claims and prescription data by calculating whether each patient receives no treatment, opioids only, therapy only or both opioid/therapy in the next visit conditioning on the state of the current visit.

© 2021 Lee B et al. *JAMA Network Open.*

eTable 7. Markov Transition Matrices From the Current Treatment Options to the Following Treatment Options Among Pain Patients in 2020

Panel A. During the pre-pandemic period.

	In the following visit				
Current visit	No treatment	Opioid only	Therapy only	Both	N
No treatment	79.73 [79.68, 79.77]	7.03 [7, 7.06]	12.08 [12.04, 12.12]	1.16 [1.15, 1.17]	2,907,380
Opioid only	50.99 [50.83, 51.15]	36.61 [36.46, 36.76]	9.39 [9.29, 9.48]	3.01 [2.96, 3.07]	372,260
Therapy only	18.75 [18.7, 18.81]	1.67 [1.65, 1.69]	77.64 [77.58, 77.7]	1.94 [1.92, 1.96]	1,851,461
Both	32.4 [32.1, 32.7]	14.33 [14.1, 14.55]	41.49 [41.18, 41.81]	11.78 [11.57, 11.99]	94,609
N	2,885,588	385,309	1,862,861	91,952	5,225,710

Panel B. During the early pandemic period.

	In the following visit				
Current visit	No treatment	Opioid only	Therapy only	Both	N
No treatment	81.03 [80.99, 81.07]	7.67 [7.64, 7.69]	10.22 [10.19, 10.25]	1.09 [1.08, 1.1]	6,860,730
Opioid only	48.32 [48.2, 48.45]	41.38 [41.25, 41.5]	7.48 [7.41, 7.54]	2.82 [2.78, 2.86]	858,438
Therapy only	19.3 [19.25, 19.36]	1.92 [1.9, 1.94]	76.69 [76.63, 76.75]	2.08 [2.06, 2.1]	2,953,523
Both	32.14 [31.87, 32.42]	15.21 [15, 15.42]	39.85 [39.57, 40.14]	12.79 [12.6, 12.99]	109,407
N	6,836,095	844,272	2,991,575	110,156	10,782,098

Note. Among pain patients who visit more than once, we measure the transition rate using weekly claims and prescription data by calculating whether each patient receives no treatment, opioids only, therapy only or both opioid/therapy in the next visit conditioning on the state of the current visit.

© 2021 Lee B et al. *JAMA Network Open.*

eTable 8. Mean Differences in Transition Rates From the Current Treatment Options to the Following Treatment Options Among Pain Patients From 2019 to 2020

Panel A. During the pre-pandemic period.

Current visit	In the following visit<br>No treatment	Opioid only	Therapy only	Both
No treatment	0.53 [0.46, 0.6]	0 [-0.04, 0.04]	-0.52 [-0.57, -0.46]	-0.01 [-0.02, 0.01]
Opioid only	-0.16 [-0.39, 0.07]	0.97 [0.75, 1.19]	-0.47 [-0.6, -0.33]	-0.34 [-0.42, -0.27]
Therapy only	0.76 [0.67, 0.85]	0.07 [0.04, 0.09]	-0.85 [-0.95, -0.75]	0.03 [0, 0.06]
Both	0.36 [-0.09, 0.8]	0.9 [0.58, 1.21]	-1.16 [-1.63, -0.7]	-0.09 [-0.43, 0.25]

Panel B. During the early pandemic period.

Current visit	In the following visit<br>No treatment	Opioid only	Therapy only	Both
No treatment	1.79 [1.73, 1.85]	0.74 [0.71, 0.78]	-2.47 [-2.51, -2.42]	-0.06 [-0.08, -0.05]
Opioid only	-2.76 [-2.94, -2.57]	5.75 [5.56, 5.94]	-2.47 [-2.57, -2.37]	-0.52 [-0.58, -0.46]
Therapy only	1.86 [1.78, 1.94]	0.36 [0.33, 0.38]	-2.38 [-2.47, -2.29]	0.17 [0.14, 0.19]
Both	0.81 [0.43, 1.18]	1.78 [1.51, 2.05]	-3.32 [-3.73, -2.92]	0.74 [0.43, 1.05]

Note. This table presents the mean difference in the transition rates from 2019 (reported in eTable 6) to 2020 (reported in eTable 7) during the same pre-pandemic and early pandemic period with 95% confidence intervals obtained from regression models that account for clustering of overlapping patients between 2019 and 2020.

© 2021 Lee B et al. *JAMA Network Open.*
